# Prevalence and Determinants of Energy Drink Consumption Among Chilean Adolescents

**DOI:** 10.3390/nu17213481

**Published:** 2025-11-05

**Authors:** Sandra López-Arana, Edson Bustos-Arriagada

**Affiliations:** Escuela de Nutrición y Dietética, Facultad de Medicina, Universidad Finis Terrae, Providencia 7501014, Chile; edsonbustos@uft.cl

**Keywords:** energy drinks, adolescents, parental monitoring, alcohol consumption, risk behaviors

## Abstract

**Background:** Energy drink (ED) consumption has increased significantly among adolescents worldwide, constituting a high-risk behavior with important public health implications. These beverages are associated with cardiovascular disturbances, sleep disorders, anxiety symptoms, and risky behaviors, especially when combined with alcohol. In Chile, monthly per capita ED consumption among individuals aged 14 to 30 increased ninefold between 2010 and 2020. **Objectives:** To examine the prevalence of ED consumption and its associated determinants among Chilean adolescents enrolled in grade eight through grade twelve. **Methods:** Data were drawn from the national representative survey 2023 entitled Fifteenth National Study on the School Population of Chile (ENPE). The final sample included 45,042 adolescents. Besides descriptive analyses, both bivariate and multivariate logistic regression models were used to examine associations between sociodemographic characteristics, parental presence, monitoring, and ED consumption outcomes. **Results:** Lifetime prevalence of ED consumption was 71.0%, with higher rates in females (72.8%) than males (69.4%). Past-month consumption was reported by 46.2%, with a higher prevalence in males (48.3%) than females (43.9%). Lifetime consumption of ED mixed with alcohol (AmED) was 23.2%, being more frequent among females (26.4%) than males (19.9%). Age, grade level, and indigenous identity were consistently associated with higher odds of consumption. Parental monitoring and involvement indicators were inversely associated with both ED and AmED consumption. **Conclusions:** This study reveals a high prevalence of ED consumption among Chilean adolescents, with notable gender differences. Family protective factors, particularly parental monitoring and cohesion, emerge as key determinants of this risky behavior and warrant prioritization in public health prevention efforts.

## 1. Introduction

Globally, the consumption of energy drinks (EDs) has increased steadily over recent decades, emerging as a high-risk behavior with significant public health implications, particularly among school-aged children, adolescents, and young adults [1,2,3]. These beverages typically contain caffeine, taurine, guarana, B-complex vitamins, and sugars, and are widely marketed by the food industry as enhancers of physical and cognitive performance [4]. Such promotion has contributed to their social acceptance and rising consumption within these populations [5,6].

Multiple studies have reported that excessive intake of EDs, defined in schoolchildren and adolescents as consumption exceeding 100 mg of caffeine per day [2,7], is associated with a range of adverse health outcomes, including cardiovascular disturbances, behavioral and sleep disorders, anxiety symptoms, reduced academic performance, and engagement in risky behaviors, especially when consumed alone or in combination with alcohol and/or other substances [5,8,9,10]. ED consumption has also been linked to unhealthy lifestyle patterns, such as lower levels of physical activity, eating disorders, and concurrent use of tobacco, alcohol, caffeine, and sugar-sweetened beverages [11,12]. Concerns regarding the safety of EDs are heightened by their high caffeine content and targeted marketing strategies aimed at youth [13,14,15].

The co-consumption of EDs with alcohol has become increasingly common and is particularly concerning, as caffeine may mask the sensation of intoxication, thereby facilitating continued alcohol intake and increasing the risk of acute alcohol poisoning and related injuries [8,16]. Additionally, a growing pattern of ED and sugar-sweetened beverage consumption has been observed among adolescents and young adults, often associated with dependence and a diminished perception of health-related harm [17,18].

In Latin America, studies report adolescent ED consumption prevalence ranging from 18% to 40% [3,19], with increasing trends observed among females and individuals experiencing lower levels of parental supervision [19]. In Chile, a 2022 study conducted by the National Corporation of Consumers and Users (CONADECUS) found that monthly per capita ED consumption among individuals aged 14 to 30 increased ninefold between 2010 and 2020, from 0.4 to 3.6 L, raising significant public health concerns. While these reports provide partial estimates of ED intake among adolescents, until now, no nationally representative studies have explored the sociocultural and familial determinants underlying this behavior.

The Fifteenth National School Population Study (ENPE) 2023, conducted by Chile’s National Service for the Prevention and Rehabilitation of Drug and Alcohol Use (SENDA), examined ED consumption behaviors among Chilean adolescents enrolled from eighth grade through to the final year of secondary education (equivalent to twelfth grade). The study explored variables related to gender, age of initiation, co-consumption with alcohol, and family factors such as parental supervision and household dynamics (https://www.senda.gob.cl/estudio-observatorio/poblacion-escolar/, accessed on 30 October 2025).

Scientific evidence has consistently shown that parental supervision and family cohesion serve as protective factors against risky behaviors in adolescence, including ED consumption [17,19]. Practices such as sharing family meals, parental awareness of adolescents’ friendships, frequented locations, and media exposure (e.g., social networks and television) are associated with lower substance use during this developmental stage [20]. However, emerging research indicates that the quality and developmental appropriateness of parental involvement are critical determinants of adolescent outcomes. Helicopter parenting, defined by excessive involvement, overcontrol, and behaviors that do not align with the adolescent’s developmental stage, has been linked to paradoxically negative outcomes in both adolescents and young adults [21,22]. Studies have documented that both high and low levels of maternal helicopter parenting are associated with a quadratic relationship to alcohol use in adolescents. Moderate levels of parental involvement appear to be the most protective, whereas extreme overinvolvement or underinvolvement are linked to increased engagement in risky behaviors [21]. Specifically, research indicates that helicopter parenting is associated with a greater likelihood of behaviors such as gambling, self-injury, illegal drug use, cigarette smoking, and problematic substance use [21,23], along with elevated rates of anxiety, depression, and reduced self-efficacy [22].

ED consumption also exhibits gender-based differences. International studies have reported that while adolescent boys tend to show higher overall prevalence of ED use [24,25], girls are more likely to initiate consumption at earlier ages and to combine EDs with alcohol more frequently [17,26]. This pattern poses additional health risks, given that adolescent girls may be more susceptible to the adverse effects of caffeine and more exposed to social pressures in recreational settings [5].

Given this context, it is essential to characterize ED consumption among Chilean adolescents and to identify the factors that influence this behavior. Such information is critical for guiding public health prevention and regulatory efforts, especially considering that Chilean legislation still presents gaps regarding the marketing, advertising, and accessibility of these products to younger populations. Accordingly, the objective of this study was to examine the prevalence of ED consumption and to assess the determinants associated with their use among Chilean adolescents enrolled from eighth grade through to the final year of secondary education, based on data from the 2023 ENPE survey conducted by SENDA.

## 2. Materials and Methods

### 2.1. Study Design and Participants

The data came from the ENPE 2023 [27], a nationally representative survey of Chilean middle and high school students in both public and private schools. This biennial study provides essential empirical data that underpin the development and implementation of evidence-based interventions targeting the prevention of alcohol and other substance use among school-aged youth.

Briefly, the sampling design was probabilistic, representative of the target population, stratified, clustered, and two-stage. The selection of the classes to be surveyed was carried out in two phases: (a) Primary Sampling Units (PSUs): defined as the classes within each stratum. PSUs were selected using probability proportional to size (PPS), where size was measured by the number of students per class. The Sampford method was employed for PSU selection [28], as an extension of Brewer’s method, which allows for the selection of more than two units per stratum, with proportional probability to size and without replacement [29]. This ensured that the final sample did not contain duplicate elements. (b) Secondary Sampling Units (SSU): defined as students within the selected classes in each stratum. SSUs were selected with equal probability.

All measures used in this study were derived from the standardized questionnaire of ENPE 2023, developed by Ipsos Chile and approved by SENDA. The questionnaire was administered using a single optical-read response sheet, designed to facilitate comprehension and maximize response rates. The full instrument, technical documentation [27], and the dataset with no identifiable information on survey participants are publicly available at (https://www.senda.gob.cl/estudio-observatorio/poblacion-escolar/, accessed on 30 October 2025).

The fieldwork was conducted between September 2023 and April 2024 by Ipsos Chile. A total of 49,211 students were surveyed, representing a population of 878,046 students across 16 regions and 134 municipalities in Chile. The overall response rate was 69.5%. By administrative dependency, the lowest response rate was observed in private schools at 56.0%. By grade level, the 4th year of high school achieved a response rate of 62.9%. In this study, we restricted our sample to adolescents who had valid data for ED consumption (*n* = 47,590). We excluded adolescents with missing values for age, gender, and variables related to parent’s relationship (*n* = 2548). The final sample included 45,042 individuals.

### 2.2. Energy Drink Consumption

Participants were asked whether they had ever consumed EDs in their lifetime, with examples provided regarding ED brands. They were also asked whether they had ever consumed EDs mixed with alcohol (AmED). Both items used a binary response format (“yes” or “no”).

To assess recent consumption, participants indicated when they last consumed EDs and AmED, selecting from four options: “during the last 30 days”, “more than a month ago, but less than a year”, “more than a year ago”, and “I have never tried.” For analytical purposes, we recategorized these into a binary variable as consumption during the last 30 days or no recent consumption.

### 2.3. Determinants of Energy Drink Consumption

The self-administered questionnaire included the following: (i) Sociodemographic characteristics (gender, sex, grade level, and belonging or not belonging to any of the nine indigenous peoples recognized in Chile). (ii) Participants were asked to indicate the individuals they currently live with, selecting from eight predefined categories (e.g., “mother and father,” “only with mother,” “only with siblings,” and “other responsible adult”). These responses were collapsed into a binary variable reflecting parental presence in the household as living with at least one parent or living without a parent. (iii) Parental monitoring and involvement explored adolescents’ perceptions through nine items. Participants were asked about the frequency with which their parent or guardian was unaware of their whereabouts after school or on weekends (e.g., for one hour or more), with response options ranging from “never or almost never” to “sometimes” and “always or almost always”. Parental awareness of media consumption was assessed with a yes/no item regarding whether parents or guardians paid attention to the television programs or internet content viewed by the adolescent. School-related involvement was measured by asking how attentive parents or guardians were to the adolescent’s school activities, with response options ranging from “a lot” to “none.” Shared mealtimes were assessed by asking how many days per week the adolescent sat at the same table with their parent or guardian for any meal (breakfast, lunch, afternoon snack, or dinner), with options ranging from “daily occurrences” to “other occurrences”. Weekend supervision was evaluated through two yes/no items: one regarding whether parents or guardians monitored the adolescent’s return time at night, and another about whether they asked or expected to be informed about the adolescent’s destination when leaving the house. Finally, participants were asked to rate how well their parent or guardian knew their closest friends, with response options of “considerably,” “somewhat,” or “barely”.

### 2.4. Statistical Analysis

Descriptive analyses were conducted to characterize the sample and estimate the weighted prevalence of four outcome variables: (i) lifetime consumption of EDs, (ii) lifetime consumption of EDs mixed with alcohol, (iii) past-month consumption of EDs, and (iv) past-month consumption of EDs mixed with alcohol. These estimates were calculated both overall and stratified by gender.

Bivariate logistic regression models were used to examine associations between each outcome and key independent variables, including sociodemographic characteristics, parental presence, monitoring, and involvement. Interaction terms were tested across all four outcomes to explore potential effect modification by gender, grade level, and other relevant covariates. Based on statistically significant interactions, stratified analyses by gender were conducted to further examine differential associations between predictors and ED consumption.

Subsequently, multivariate logistic regression models were estimated separately for female and male adolescents. Each model included all independent variables adjusted for age, residence, and ethnicity. This approach allowed for the identification of independent associations between each determinant and the four outcomes of interest, while accounting for potential confounding by sociodemographic factors.

To assess the impact of excluding cases with missing data, we conducted a sensitivity analysis comparing prevalence estimates and consumption patterns between the complete case sample and the full sample. The test of homogeneity was used to evaluate differences across key outcome variables. Results are summarized in Supplementary Table S1 and indicate consistent findings across samples.

All analyses incorporated survey sample weights using Stata’s *svy* commands to account for the complex sampling design. Weighted percentages, odds ratios (ORs), 95% confidence intervals (95% CIs), and *p*-values were reported. Statistical significance was defined as *p* < 0.05. Analyses were performed using Stata version 17 (StataCorp, College Station, TX, USA).

### 2.5. Ethical Considerations

The protocol of this study was reviewed and approved with a waiver of informed consent by the Ethics Committee of the Universidad Finis Terrae (protocol ID: 25-055; approval date: 30 June 2025; plenary session n◦10).

## 3. Results

### 3.1. Sample Characteristics

Table 1 presents the sociodemographic characteristics of adolescent participants (*n* = 45,042). Females represented 48.5% of the sample, while males accounted for 51.5%. The sample included 36.1% of participants living in the metropolitan region and 63.9% in other regions, and participant distribution by grade level varied slightly, with percentages ranging from 17.8% to 21.7%.

Approximately 16.1% of adolescents self-identified as belonging to an indigenous group, while 48.1% reported non-Indigenous identity and 35.8% were uncertain or preferred not to answer. Most participants lived with at least one parent (85.2%), and a substantial proportion reported frequent shared meals with family members (55.0%, ≥5 times per week).

Parental monitoring varied across domains. While 44.7% of adolescents reported that their parents were aware of their media use, a substantially higher proportion (94.8%) indicated consistent parental knowledge of their social outings. In terms of school-related activities, 28.0% of participants reported high parental involvement, and 48.0% described it as moderate, highlighting differences in engagement across contexts.

### 3.2. Consumption of Energy Drinks or EDs Mixed with Alcohol

**Figure 1:**
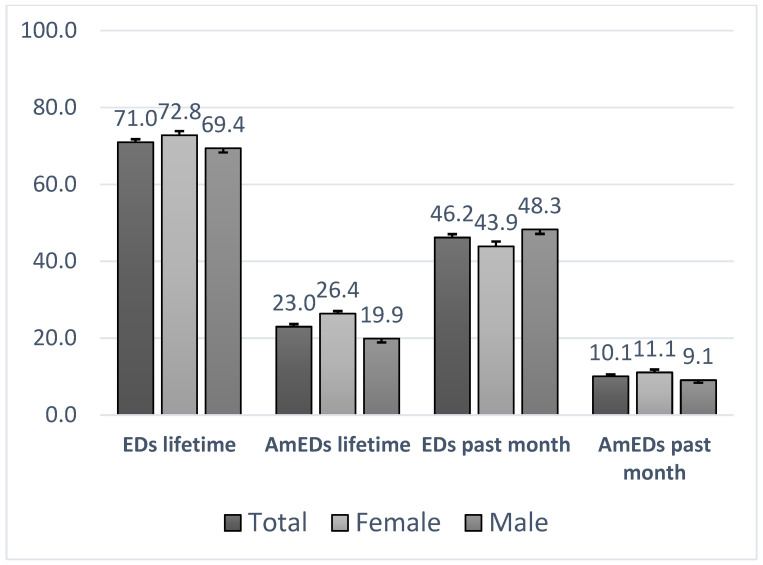
Energy drink consumption: lifetime, past-month, and mixed use with alcohol by gender and total sample. EDs: energy drinks; AmEDs: energy drinks mixed with alcohol.

Among the Chilean adolescents surveyed, 71.0% reported lifetime consumption of EDs, with a slightly higher prevalence among females (72.8%) compared to males (69.4%). Past-month ED use was reported by 46.2% of participants, with males exhibiting higher recent use (48.3%) than females (43.9%). Lifetime consumption of energy drinks mixed with alcohol (AmEDs) was reported by 23.2% of adolescents, with a greater proportion among females (26.4%) than males (19.9%). Past-month AmED use was less frequent overall (10.1%), but again more prevalent among females (11.1%) than males (9.1%) (Figure 1).

### 3.3. Associations Between Sociodemographic Characteristics; Parental Presence, Monitoring, and Involvement; and Consumption of Energy Drinks or EDs Mixed with Alcohol

Table 2 displays the results of bivariate logistic regression analyses examining the association between individual sociodemographic and parental factors and ED consumption outcomes. Each predictor was modeled separately, without adjustment for other covariates.

Being female was positively associated with lifetime ED consumption (OR: 1.18; 95% CI: 1.08–1.27) and lifetime consumption of energy drinks mixed with alcohol (AmEDs) (OR: 1.44; 95% CI: 1.33–1.56) but inversely associated with past-month ED use (OR: 0.84; 95% CI: 0.79–0.90).

Age and grade level were consistently associated with greater odds of all consumption outcomes. For example, the 4th year of high school students had significantly higher odds of lifetime AmED use (OR: 3.24; 95% CI: 2.85–3.69) and past-month AmED use (OR: 2.22; 95% CI: 1.85–2.66) compared to the reference category.

Ethnic identity was differentially associated with ED and AmED use. Those who did not identify as indigenous had significantly higher odds of lifetime ED use (OR: 1.26; 95% CI: 1.14–1.41) and lower odds of AmED use in the past month (OR: 0.78; 95% CI: 0.67–0.90). Adolescents who were uncertain about their ethnic identity had significantly lower odds of lifetime ED use (OR: 0.89; 95% CI: 0.81–0.99) and AmED use in the past month (OR: 0.79; 95% CI: 0.67–0.93).

Parental monitoring and engagement indicators were inversely associated with ED and AmED consumption. Lower parental awareness of media use, curfew adherence, and social outings were linked to higher odds. For instance, adolescents whose parents were unaware of their social outings had higher odds of past-month AmED use. Similarly, infrequent shared meals and limited parental involvement in school activities were associated with higher consumption.

Living without a parent was associated with lower odds of lifetime ED use (OR: 0.63; 95% CI: 0.57–0.70), though no significant associations were observed for AmED outcomes.

Table 3 and Table 4 present the bivariate and multivariate logistic regression analyses examining the association between sociodemographic and parental factors and ED consumption outcomes among males. Age was consistently associated with higher odds of both ED and ED mixed with alcohol consumption. The grade level showed a dose–response relationship; 4th year of high school males had markedly higher likelihood of past-month ED combined with alcohol consumption (OR = 4.04, 95% CI: 3.05–5.36) in the unadjusted model. This association was attenuated but remained statistically significant (OR = 2.51, 95% CI: 1.59–3.16). Male adolescents whose parents always knew their location had lower odds of consumption, while lack of monitoring was associated with higher odds. Parental awareness of school activities and close friends showed a positive association, with lower awareness linked to higher consumption.

Table 5 and Table 6 outline associations between sociodemographic and parental factors and ED consumption among female adolescents. Overall age was positively associated with lifetime ED and ED mixed with alcohol consumption and consumption of EDs combined with alcohol in the past month. Grade level was a strong determinant of ED and AmED consumption, but less attenuated than in male adolescents. Females whose parents or guardians sometimes knew their location had significantly higher odds of ED and AmED consumption compared to those whose parents always or almost always knew their location. Following similar patterns to males, lower parental awareness of school activities and close friends were associated with higher likelihood of consumption.

## 4. Discussion

This secondary analysis based on the 2023 Fifteenth National Study on the School Population of Chile examined the prevalence and determinants of ED and AmED consumption among Chilean adolescents, with a particular focus on gender differences. The findings reveal high rates of lifetime and past-month ED use, and a concerning prevalence of AmED consumption, particularly among older students. Across both genders, parental monitoring and involvement emerged as critical protective factors. Adolescents whose parents consistently knew their location, expected disclosure of outings, and were aware of school activities and friendships were significantly less likely to consume EDs and AmEDs. These results underscore the importance of engaged parenting, not only in setting boundaries, but also in fostering open communication and relational trust, as a buffer against risky consumption behaviors.

The prevalence of ED consumption among Chilean adolescents appears to be high compared to global estimates. In this study, 71.0% reported lifetime ED consumption, while 46.2% reported recent use in the past month. These figures exceed the worldwide pooled prevalence reported by Aonso-Diego et al. (2024), which estimated lifetime ED use at 54.7% and past-month use at 32.3% among adolescents and young adults [3]. In contrast, the prevalence of AmED consumption in Chile was relatively lower. Lifetime AmED use was reported by 23.2% of adolescents, and past month use by 10.1%. These rates fall below European estimates from Scalese et al. (2024), where 33.9% of 16-year-olds reported AmED use in the past year, with national rates ranging from 14.9% in Latvia to 53.7% in Slovenia [30]. Notably, while European data showed higher prevalence among males (37.3%) than females (30.6%), the Chilean sample revealed the opposite pattern; females informed greater lifetime (26.4%) and past-month (11.1%) AmED use than males (19.9% and 9.1%, respectively).

The gendered patterns observed in this study may reflect distinct mechanisms of initiation and normalization. Among male adolescents, age and grade level were strongly associated with increased odds of both ED and AmED consumption, suggesting a progressive normalization of these behaviors as adolescents advance through school. On the other hand, the higher prevalence among females, particularly in lifetime AmED use, may point to earlier experimentation or relational contexts that facilitate initial exposure. Prior research has shown that female adolescents may engage in risk behaviors earlier, often in peer-driven or socially embedded settings, while male patterns tend to intensify with age and autonomy [31,32]. Longitudinal evidence further supports this differentiation. Brunborg et al. (2022) found that the effect of ED use on concurrent alcohol consumption was stronger among girls, whereas the increase in use over time was more pronounced among boys, suggesting gender-specific trajectories in substance co-use [26]. Additionally, qualitative research has highlighted how ED marketing has traditionally aligned with masculinity norms, but adolescents now perceive certain products as gender-targeted, with packaging and branding increasingly appealing to both male and female consumers [33]. This shift may help explain the rising prevalence of ED use among girls, as observed in Finnish adolescents, where weekly consumption has increased considerably, indicating a possible erosion of gender-normative health behaviors [17].

Parental monitoring, particularly consistent knowledge of adolescents’ whereabouts and expectations around disclosure of outings, emerged as a protective factor across both genders. These findings align with prior research indicating that structured parental oversight can buffer against risky behaviors, especially among male adolescents [19,34]. For females, however, parental factors appeared even more influential. Although associations between age and energy drink (ED) use were slightly weaker among girls, lack of parental awareness regarding school activities and close friendships was strongly linked to alcohol mixed with energy drink (AmED) consumption. This suggests that relational and communicative dimensions of parenting such as emotional connection and attentiveness to adolescents’ social contexts may play a particularly critical role in shaping female health behaviors. The observed patterns can be better understood by integrating multiple adolescent development theories. For example, Family Systems Theory posits that individual behaviors occur within broader relational networks; thus, parental monitoring reflects system quality, where open communication and relational trust serve as buffers against risk behaviors [34,35]. Likewise, Social Control Theory emphasizes that strong social bonds, attachment, commitment, involvement, and belief in societal rules might help prevent adolescents from engaging in deviant behavior. Within this framework, parental monitoring acts as a direct preventive mechanism. Adolescents may refrain from substance use not because they fear punishment, but because they anticipate parental detection and wish to maintain trust within the relationship [34]. Finally, the bidirectional model of adolescent self-regulation suggests that high-quality relationships foster self-regulatory capacities, which in turn support healthier decision-making and resistance to external pressures such as ED and AmED consumption [36]. Based on this theoretical framework, the high prevalence of AmED consumption, especially among older adolescents, raises significant public health concerns. AmEDs combine stimulants and depressants, which can mask intoxication and increase the risk of binge drinking, accidents, and other adverse outcomes [16]. The strong associations with low parental monitoring and weak curfew enforcement suggest that AmED use may be embedded within broader patterns of unsupervised socialization and risk-taking. While consistent parental monitoring emerged as protective, it is important to acknowledge that the “always or almost always” response category may encompass a range of parenting styles, including potentially overinvolved or controlling behaviors. In some contexts, this has been referred to as helicopter parenting [21,22,23], which may inadvertently undermine adolescent autonomy. Although our study did not directly assess parenting style, future research could explore whether high-frequency monitoring reflects relational trust or excessive oversight, and how these nuances may differentially impact adolescent health behaviors.

Taken together, these findings underscore the need for gender-sensitive public health strategies. For male adolescents, interventions might focus on delaying initiation and reinforcing parental boundaries during key transitional school years. For females, programs that strengthen parent–child communication and emotional engagement may be more effective. School-based education should address the specific risks associated with AmEDs, not just EDs, and incorporate components that actively involve families in prevention efforts.

Although this study draws on a nationally representative survey, lending strength to its generalizability and external validity, several limitations should be acknowledged. First, the data rely on adolescent self-reports, which may be subject to recall and social desirability biases. It is possible that participants underestimated their frequency of ED consumption. Prior research has shown that underreporting is more pronounced for substances perceived as socially unacceptable (e.g., cocaine and opiates) than for more normalized substances such as cannabis [37,38]. Moreover, inconsistencies between self-reported use and biological verification have been documented, with some adolescents reporting substance use despite negative urinalysis results, further obscuring prevalence estimates [38]. Second, the cross-sectional design limits the ability to draw causal inferences. While this study identifies associations between parental monitoring and consumption behaviors, it cannot determine the directionality or temporal dynamics of these relationships. Future longitudinal research is needed to explore how parental involvement evolves over time and interacts with peer influences and developmental trajectories. Third, qualitative studies could provide deeper insight into the motivations behind AmED use, particularly in relation to gendered social norms, marketing strategies, and adolescent perceptions of risk. Understanding these contextual factors may inform more targeted interventions. Fourth, although the ENPE questionnaire was designed to be clear and easy to complete, formal validation studies of individual items are not publicly available. However, the instrument has been used for over a decade in national surveys, was approved by SENDA, and was implemented through a standardized protocol. It is considered a reliable tool for monitoring adolescent substance use in Chile. Finally, the findings underscore the need for comprehensive public health strategies to reduce ED consumption among adolescents. Policy measures could include increasing the price of EDs, establishing a minimum legal age for purchase, mandating transparent ingredient labeling and warnings about potential adverse effects, and regulating advertising, sponsorship, and promotional practices aimed at youth.

## 5. Conclusions

This study contributes to a growing body of evidence on ED and AmED consumption among adolescents, highlighting important gender differences, developmental patterns, and the protective role of parental involvement. Drawing on nationally representative data from Chile, the findings reveal high rates of ED use and a concerning prevalence of AmED consumption, particularly among older students and female adolescents. These patterns suggest shifting gender norms and underscore the need for culturally responsive prevention strategies. Importantly, this study emphasizes that engaged parenting, characterized by consistent monitoring, open communication, and relational trust remains a key buffer against risky consumption behaviors. Future research and policy efforts should build on these insights to promote adolescent health through both structural regulation and family-based interventions.

## Figures and Tables

**Table 1:** Characteristics of the study population (unweighted sample *n* = 45,042).

Variable	*n* (Weighted %) Median (Weighted 95% CI)
**Gender**	
Male	23,228 (51.5)
Female	21,814 (48.5)
**Age (years)**	16.0 (15.5–16.5)
**Grade**	
8th grade	8863 (19.9)
1st year of high school	9494 (21.7)
2nd year of high school	9859 (20.6)
3rd year of high school	9130 (20.0)
4th year of high school	7696 (17.8)
**Ethnicity (Belonging to Any of the Nine Indigenous Peoples)**	
Yes	7664 (16.1)
No	20,831 (48.1)
Do not know	16,547 (35.8)
**Residence**	
Metropolitan region	12,738 (36.1)
Another region	32,304 (63.9)
**Parent’s Presence**	
Living with at least one parent	38,006 (85.2)
Living without parent	7036 (14.8)
**Parent/Guardian Knowledge of Location**	
Never or almost never know	2659 (5.3)
Sometimes know	7396 (15.9)
Always or almost always know	34,987 (78.8)
**Parental Monitoring of Media Use**	
Yes	19,799 (44.7)
No	25,243 (55.3)
**Parental Awareness of School Activities**	
A lot	12,539 (28.0)
Fairly	21,543 (48.0)
A little	9699 (21.5)
None	1261 (2.5)
**Frequency of Shared Meals with Parent/Guardian**	
Daily occurrences	24,702 (55.0)
Other occurrences	20,340 (45.0)
**Weekend Curfew Monitoring by Parent/Guardian**	
Yes	37,056 (83.4)
No	7986 (16.6)
**Parental Expectation to Disclose Outings**	
Yes	42,079 (94.8)
No	2963 (5.2)
**Parental Awareness of Close Friends**	
Considerable	18,973 (41.4)
Somewhat	19,589 (44.0)
Barely	6480 (14.6)

95% CI: 95% confidence interval.

**Table 2:** Bivariate logistic regression of sociodemographic and parental determinants of energy drink consumption and consumption mixed with alcohol over one’s lifetime and in past month.

Variable	EDs Lifetime OR (95% CI)	AmEDs Lifetime OR (95% CI)	EDs Past Month OR (95% CI)	AmEDs Past Month OR (95% CI)
**Gender**				
Male	(Reference)	(Reference)	(Reference)	(Reference)
Female	1.18 (1.09–1.27) **	1.44 (1.33–1.56) **	0.84 (0.79–0.90) **	1.26 (1.12–1.41) **
**Age**	1.14 (1.11–1.17) **	1.28 (1.25–1.32) **	1.08 (1.06–1.11) **	1.19 (1.15–1.24) **
**Ethnicity**				
Yes	(Reference)	(Reference)	(Reference)	(Reference)
No	1.26 (1.14–1.41) **	0.92 (0.83–1.03)	1.07 (0.97–1.18)	0.78 (0.67–0.90) **
Do not know	0.89 (0.81–0.99) *	0.79 (0.71–0.89) **	0.93 (0.84–1.02)	0.79 (0.67–0.93) **
**Grade**				
8 basic	(Reference)	(Reference)	(Reference)	(Reference)
1st year of high school	1.33 (1.19–1.49) **	1.64 (1.42–1.89) **	1.32 (1.19–1.47) **	1.40 (1.15–1.71) **
2nd year of high school	1.71 (1.54–1.90) **	2.17 (1.91–2.46) **	1.51 (1.37–1.66) **	1.60 (1.34–1.91) **
3rd year of high school	1.92 (1.73–2.15) **	3.02 (2.66–3.42) **	1.56 (1.41–1.73) **	2.16 (1.81–2.57) **
4th year of high school	1.88 (1.67–2.12) **	3.24 (2.85–3.69) **	1.53 (1.37–1.70) **	2.22 (1.85–2.66) **
**Residence**				
Metropolitan region	(Reference)	(Reference)	(Reference)	(Reference)
Other region	0.98 (0.90–1.06)	0.95 (0.87–1.04)	0.95 (0.88–1.03)	0.90 (0.79–1.03)
**Parent’s presence**				
Living with at least one parent	(Reference)	(Reference)	(Reference)	(Reference)
Living without parent	0.63 (0.57–0.70) **	0.94 (0.84–1.05)	0.79 (0.72–0.87) **	1.06 (0.90–1.24)
**Parent/guardian knowledge of location**				
Always or almost always know	(Reference)	(Reference)	(Reference)	(Reference)
Sometimes	1.70 (1.53–1.89) **	2.12 (1.92–2.34) **	1.80 (1.64–1.97) **	2.21 (1.94–2.52) **
Never or almost never know	1.04 (0.88–1.23)	1.44 (1.23–1.69) **	1.12 (0.96–1.29)	1.57 (1.26–1.94) **
**Parental monitoring of media use**				
Yes	(Reference)	(Reference)	(Reference)	(Reference)
No	1.60 (1.48–1.72) **	1.62 (1.49–1.76) **	1.45 (1.36–1.56) **	1.54 (1.37–1.73) **
**Parental awareness of school activities**				
A lot	(Reference)	(Reference)	(Reference)	(Reference)
Fairly	1.55 (1.42–1.69) **	1.31 (1.19–1.45) **	1.27 (1.17–1.37) **	1.37 (1.19–1.59) **
A little	1.87 (1.68–2.08) **	1.98 (1.77–2.22) **	1.73 (1.56–1.90) **	1.97 (1.67–2.31) **
None	1.25 (0.99–1.58)	2.44 (1.92–3.09) **	1.19 (0.96–1.49)	2.18 (1.66–2.88) **
**Shared meals with parent/guardian**				
Daily occurrences	(Reference)	(Reference)	(Reference)	(Reference)
Other occurrences	1.43 (1.32–1.54) **	1.60 (1.48–1.73) **	1.47 (1.37–1.58) **	1.56 (1.40–1.75) **
**Weekend curfew monitoring by parent/guardian**				
Yes	(Reference)	(Reference)	(Reference)	(Reference)
No	0.90 (0.82–0.99) *	1.42 (1.28–1.57) **	1.08 (0.99–1.18)	1.60 (1.40–1.84) **
**Parental expectation to disclose outings**				
Yes	(Reference)	(Reference)	(Reference)	(Reference)
No	0.61 (0.52–0.70) **	1.53 (1.31–1.78) **	0.89 (0.77–1.02)	1.49 (1.23–1.79) **
**Parental awareness of close friends**				
Considerable	(Reference)	(Reference)	(Reference)	(Reference)
Somewhat	1.24 (1.15–1.34) **	1.19 (1.09–1.29) **	1.16 (1.08–1.25) **	1.13 (0.99–1.27)
Barely	1.35 (1.21–1.51) **	1.46 (1.30–1.64) **	1.32 (1.19–1.46) **	1.57 (1.33–1.85) **

EDs: energy drinks; AmEDs: energy drinks mixed with alcohol; OR: odds ratio; 95% CI: 95% confidence interval; * *p*-value: <0.05; ** *p*-value: <0.001.

**Table 3:** Bivariate logistic regression of sociodemographic and parental determinants of energy drink consumption and consumption of energy drinks mixed with alcohol over one’s lifetime and in past month among males.

Variable	EDs Lifetime OR (95% CI)	AmEDs Lifetime OR (95% CI)	EDs Past Month OR (95% CI)	AmEDs Past Month OR (95% CI)
**Age**	1.15 (1.12–1.20) **	1.36 (1.31–1.42) **	1.14 (1.10–1.17) **	1.30 (1.23–1.37) **
**Ethnicity**				
Yes	(Reference)	(Reference)	(Reference)	(Reference)
No	1.28 (1.10–1.47) **	0.88 (0.75–1.04)	1.15 (1.01–1.32) **	0.74 (0.60–0.92) *
Do not know	0.90 (0.79–1.04)	0.81 (0.68–0.95) *	0.88 (0.77–1.00)	0.79 (0.63–0.99)
**Grade**				
8 basic	(Reference)	(Reference)	(Reference)	(Reference)
1st year of high school	1.43 (1.23–1.66) **	1.98 (1.56–2.51) **	1.44 (1.24–1.67) **	1.94 (1.41–2.68) **
2nd year of high school	1.80 (1.55–2.08) **	2.66 (2.18–3.24) **	1.57 (1.37–1.80) **	2.25 (1.70–2.96) **
3rd year of high school	1.84 (1.58–2.15) **	3.71 (3.04–4.52) **	1.78 (1.54–2.05) **	3.07 (2.30–4.08) **
4th year of high school	2.12 (1.80–2.50) **	4.74 (3.88–5.78) **	1.98 (1.71–2.29) **	4.04 (3.05–5.36) **
**Residence**				
Metropolitan region	(Reference)	(Reference)	(Reference)	(Reference)
Other region	0.99 (0.88–1.11)	1.00 (0.87–1.14)	0.99 (0.88–1.10)	0.86 (0.71–1.03)
**Parent’s presence**				
Living with at least one parent	(Reference)	(Reference)	(Reference)	(Reference)
Living without parent	0.62 (0.54–0.70) **	0.95 (0.80–1.11)	0.78 (0.69–0.89) **	1.06 (0.84–1.34)
**Parent/guardian knowledge of location**				
Always or almost always know	(Reference)	(Reference)	(Reference)	(Reference)
Sometimes	1.69 (1.48–1.94) **	2.16 (1.89–2.47) **	1.61 (1.43–1.81) **	2.16 (1.81–2.57) **
Never or almost never know	1.01 (0.81–1.27)	1.60 (1.28–2.00) **	1.09 (0.89–1.33)	1.85 (1.37–2.51) **
**Parental monitoring of media use**				
Yes	(Reference)	(Reference)	(Reference)	(Reference)
No	1.61 (1.46–1.79) **	1.77 (1.55–2.03) **	1.39 (1.26–1.53) **	1.53 (1.27–1.84) **
**Parental awareness of school activities**				
A lot	(Reference)	(Reference)	(Reference)	(Reference)
Fairly	1.50 (1.34–1.68) **	1.27 (1.09–1.48) *	1.16 (1.04–1.29) *	1.20 (0.97–1.49)
A little	1.68 (1.45–1.95) **	2.01 (1.69–2.40) **	1.54 (1.34–1.76) **	1.75 (1.38–2.23) **
None	1.01 (0.72–1.41)	2.15 (1.47–3.13) **	1.08 (0.78–1.49)	1.64 (1.07–2.51) *
**Shared meals with parent/guardian**				
Daily occurrences	(Reference)	(Reference)	(Reference)	(Reference)
Other occurrences	1.33 (1.20–1.47) **	1.60 (1.42–1.80) **	1.45 (1.32–1.59) **	1.48 (1.26–1.75) **
**Weekend curfew monitoring by parent/guardian**				
Yes	(Reference)	(Reference)	(Reference)	(Reference)
No	0.92 (0.81–1.05)	1.54 (1.34–1.77) **	1.06 (0.94–1.19)	1.66 (1.38–2.01) **
**Parental expectation to disclose outings**				
Yes	(Reference)	(Reference)	(Reference)	(Reference)
No	0.63 (0.53–0.75) **	1.70 (1.40–2.07) **	0.86 (0.73–1.03)	1.63 (1.27–2.09) **
**Parental awareness of close friends**				
Considerable	(Reference)	(Reference)	(Reference)	(Reference)
Somewhat	1.26 (1.13–1.41) **	1.14 (1.00–1.29)	1.13 (1.02–1.26) *	0.96 (0.80–1.14)
Barely	1.45 (1.25–1.69) **	1.53 (1.30–1.81) **	1.27 (1.11–1.46) **	1.52 (1.20–1.94) *

EDs: energy drinks; AmEDs: energy drinks mixed with alcohol; OR: odds ratio; 95% CI: 95% confidence interval; * *p*-value: <0.05; ** *p*-value: <0.001.

**Table 4:** Multivariate logistic regression of sociodemographic and parental determinants of energy drinks consumption and consumption of energy drinks mixed with alcohol over one’s lifetime and in past month among males.

Variable	EDs Lifetime OR (95% CI)	AmEDs Lifetime OR (95% CI)	EDs Past Month OR (95% CI)	AmEDs Past Month OR (95% CI)
**Age**	1.15 (1.11–1.18) **	1.36 (1.31–1.42) **	1.13 (1.09–1.16) **	1.30 (1.24–1.38) **
**Ethnicity**				
Yes	(Reference)	(Reference)	(Reference)	(Reference)
No	1.27 (1.10–1.46) *	0.86 (0.73–1.01)	1.15 (1.00–1.31) *	0.74 (0.59–0.92) *
Do not know	0.94 (0.81–1.08)	0.87 (0.73–1.03)	0.91 (0.79–1.04)	0.86 (0.68–1.08)
**Grade**				
8 basic	(Reference)	(Reference)	(Reference)	(Reference)
1st year of high school	1.39 (1.18–1.64) **	1.70 (1.31–2.20) **	1.39 (1.18–1.63) **	1.73 (1.24–2.43) *
2nd year of high school	1.69 (1.38–2.07) **	1.95 (1.53–2.50) **	1.45 (1.21–1.75) **	1.77 (1.28–2.47) *
3rd year of high school	1.69 (1.30–2.19) **	2.32 (1.72–3.13) **	1.60 (1.27–2.01) **	2.12 (1.45–3.09) **
4th year of high school	1.88 (1.37–2.60) **	2.56 (1.80–3.65) **	1.71 (1.30–2.27) **	2.51 (1.59–3.96) **
**Residence**				
Metropolitan region	(Reference)	(Reference)	(Reference)	(Reference)
Other region	0.99 (0.88–1.12)	1.03 (0.90–1.19)	1.00 (0.89–1.11)	0.89 (0.74–1.06)
**Parent’s presence**				
Living with at least one parent	(Reference)	(Reference)	(Reference)	(Reference)
Living without parent	0.63 (0.55–0.72) **	0.93 (0.78–1.10)	0.81 (0.71–0.92) *	1.02 (0.81–1.29)
**Parent/guardian knowledge of location**				
Always or almost always know	(Reference)	(Reference)	(Reference)	(Reference)
Sometimes	1.69 (1.48–1.94) **	2.12 (1.84–2.44) **	1.61 (1.42–1.81) **	2.08 (1.74–2.49) **
Never or almost never know	0.98 (0.78–1.24)	1.51 (1.20–1.89) **	1.06 (0.87–1.30)	1.75 (1.29–2.38) **
**Parental monitoring of media use**				
Yes	(Reference)	(Reference)	(Reference)	(Reference)
No	1.54 (1.39–1.72) **	1.62 (1.41–1.86) **	1.33 (1.21–1.47) **	1.40 (1.17–1.69) **
**Parental awareness of school activities**				
A lot	(Reference)	(Reference)	(Reference)	(Reference)
Fairly	1.48 (1.32–1.67) **	1.23 (1.05–1.44) *	1.14 (1.02–1.27) *	1.17 (0.94–1.45)
A little	1.64 (1.41–1.90) **	1.90 (1.59–2.28) **	1.50 (1.30–1.72) **	1.65 (1.29–2.10) **
None	0.97 (0.69–1.37)	1.85 (1.26–2.73) *	1.04 (0.75–1.44)	1.40 (0.90–2.16)
**Shared meals with parent/guardian**				
Daily occurrences	(Reference)	(Reference)	(Reference)	(Reference)
Other occurrences	1.28 (1.15–1.42) **	1.48 (1.31–1.68) **	1.41 (1.28–1.55) **	1.39 (1.18–1.64) **
**Weekend curfew monitoring by parent/guardian**				
Yes	(Reference)	(Reference)	(Reference)	(Reference)
No	0.87 (0.77–0.99) *	1.33 (1.16–1.53) **	1.01 (0.90–1.13)	1.46 (1.20–1.76) **
**Parental expectation to disclose outings**				
Yes	(Reference)	(Reference)	(Reference)	(Reference)
No	0.64 (0.53–0.76) **	1.64 (1.34–2.01) **	0.87 (0.73–1.03)	1.52 (1.18–1.98) **
**Parental awareness of close friends**				
Considerable	(Reference)	(Reference)	(Reference)	(Reference)
Somewhat	1.26 (1.13–1.41) **	1.14 (1.00–1.30)	1.14 (1.03–1.27) *	0.94 (0.79–1.12)
Barely	1.45 (1.25–1.69) **	1.44 (1.22–1.71) **	1.26 (1.09–1.45) *	1.42 (1.12–1.81) *

EDs: energy drinks; AmEDs: energy drinks mixed with alcohol; OR: odds ratio; 95% CI: 95% confidence interval; * *p*-value: <0.05; ** *p*-value: <0.001. Models were adjusted for age, residence, and ethnicity.

**Table 5:** Bivariate logistic regression of sociodemographic and parental determinants of energy drink consumption and consumption of energy drinks mixed with alcohol over one’s lifetime and in past month among females.

Variable	EDs Lifetime OR (95% CI)	AmEDs Lifetime OR (95% CI)	EDs Past Month OR (95% CI)	AmEDs Past Month OR (95% CI)
**Age**	1.13 (1.09–1.18) **	1.22 (1.18–1.27) **	1.02 (0.99–1.06)	1.11 (1.04–1.17) *
**Ethnicity**				
Yes	(Reference)	(Reference)	(Reference)	(Reference)
No	1.24 (1.07–1.45) **	0.88 (0.75–1.04)	1.01 (0.87–1.16)	0.79 (0.64–0.98)
Do not know	0.90 (0.77–1.05)	0.81 (0.68–0.95) *	0.97 (0.84–1.13)	0.81 (0.65–1.01)
**Grade**				
8 basic	(Reference)	(Reference)	(Reference)	(Reference)
1st year of high school	1.25 (1.06–1.47) **	1.51 (1.26–1.80) **	1.20 (1.03–1.39) *	1.17 (0.91–1.51)
2nd year of high school	1.65 (1.42–1.92) **	1.96 (1.66–2.32) **	1.45 (1.26–1.66) **	1.32 (1.04–1.68) *
3rd year of high school	2.03 (1.74–2.38) **	2.66 (2.25–3.15) **	1.37 (1.19–1.59) **	1.73 (1.38–2.18) **
4th year of high school	1.66 (1.39–1.97) **	2.45 (2.05–2.92) **	1.16 (0.99–1.37)	1.39 (1.08–1.78) *
**Residence**				
Metropolitan region	(Reference)	(Reference)	(Reference)	(Reference)
Other region	0.97 (0.85–1.09)	0.91 (0.80–1.03)	0.92 (0.82–1.03)	0.94 (0.78–1.12)
**Parent’s presence**				
Living with at least one parent	(Reference)	(Reference)	(Reference)	(Reference)
Living without parent	0.66 (0.57–0.77) **	0.97 (0.83–1.13)	0.78 (0.68–0.89) *	1.08 (0.87–1.34)
**Parent/guardian knowledge of location**				
Always or almost always know	(Reference)	(Reference)	(Reference)	(Reference)
Sometimes	1.84 (1.55–2.20) **	2.42 (2.07–2.83) **	2.04 (1.75–2.37) **	2.51 (2.06–3.06) **
Never or almost never know	1.15 (0.91–1.46)	1.42 (1.13–1.79) **	1.10 (0.88–1.38)	1.35 (1.01–1.81) *
**Parental monitoring of media use**				
Yes	(Reference)	(Reference)	(Reference)	(Reference)
No	1.65 (1.48–1.83) **	1.61 (1.44–1.80) **	1.49 (1.35–1.64) **	1.61 (1.38–1.89) **
**Parental awareness of school activities**				
A lot	(Reference)	(Reference)	(Reference)	(Reference)
Fairly	1.60 (1.41–1.81) **	1.35 (1.18–1.54) **	1.41 (1.25–1.58) **	1.56 (1.27–1.90) **
A little	2.11 (1.81–2.47) **	1.95 (1.68–2.27) **	1.98 (1.72–2.27) **	2.19 (1.76–2.72) **
None	1.63 (1.19–2.24) **	2.77 (2.05–3.76) **	1.34 (1.00–1.80)	2.81 (1.95–4.04) **
**Shared meals with parent/guardian**				
Daily occurrences	(Reference)	(Reference)	(Reference)	(Reference)
Other occurrences	1.55 (1.39–1.74) **	1.62 (1.45–1.80) **	1.49 (1.35–1.65) **	1.64 (1.41–1.91) **
**Weekend curfew monitoring by parent/guardian**				
Yes	(Reference)	(Reference)	(Reference)	(Reference)
No	0.92 (0.78–1.07)	1.46 (1.26–1.70) **	1.05 (0.91–1.21)	1.68 (1.38–2.04) **
**Parental expectation to disclose outings**				
Yes	(Reference)	(Reference)	(Reference)	(Reference)
No	0.60 (0.47–0.76) **	1.51 (1.17–1.94) *	0.86 (0.67–1.10)	1.42 (1.08–1.87) *
**Parental awareness of close friends**				
Considerable	(Reference)	(Reference)	(Reference)	(Reference)
Somewhat	1.25 (1.11–1.40) **	1.28 (1.14–1.43) **	1.17 (1.05–1.30) *	1.32 (1.12–1.56) *
Barely	1.29 (1.09–1.53) **	1.53 (1.30–1.80) **	1.32 (1.12–1.54) **	1.68 (1.35–2.09) **

EDs: energy drinks; AmEDs: energy drinks mixed with alcohol; OR: odds ratio; 95% CI: 95% confidence interval; * *p*-value: <0.05; ** *p*-value: <0.001.

**Table 6:** Multivariate logistic regression of sociodemographic and parental determinants of energy drink consumption and consumption of energy drinks mixed with alcohol over one’s lifetime and in past month among females.

Variable	EDs Lifetime OR (95% CI)	AmEDs Lifetime OR (95% CI)	EDs Past Month OR (95% CI)	AmEDs Past Month OR (95% CI)
**Age**	1.13 (1.08–1.17) **	1.22 (1.18–1.27) **	1.02 (0.99–1.06)	1.11 (1.04–1.18) *
**Ethnicity**				
Yes	(Reference)	(Reference)	(Reference)	(Reference)
No	1.24 (1.07–1.44) *	0.92 (0.79–1.08)	1.01 (0.88–1.17)	0.79 (0.64–0.97) *
Do not know	0.94 (0.80–1.10)	0.88 (0.74–1.03)	0.99 (0.85–1.15)	0.84 (0.68–1.05)
**Grade**				
8 basic	(Reference)	(Reference)	(Reference)	(Reference)
1st year of high school	1.27 (1.06–1.51) *	1.43 (1.17–1.75) **	1.29 (1.09–1.53) *	1.11 (0.82–1.51)
2nd year of high school	1.71 (1.38–2.11) **	1.78 (1.38–2.31) **	1.66 (1.36–2.04) **	1.18 (0.76–1.85)
3rd year of high school	2.16 (1.64–2.83) **	2.31 (1.69–3.17) **	1.71 (1.32–2.22) **	1.48 (0.87–2.50)
4th year of high school	1.77 (1.26–2.49) **	2.03 (1.37–3.01) **	1.56 (1.13–2.15) *	1.13 (0.58–2.20)
**Residence**				
Metropolitan region	(Reference)	(Reference)	(Reference)	(Reference)
Other region	0.94 (0.83–1.07)	0.87 (0.77–0.99) *	0.92 (0.82–1.03)	0.92 (0.77–1.11)
**Parent’s presence**				
Living with at least one parent	(Reference)	(Reference)	(Reference)	(Reference)
Living without parent	0.68 (0.58–0.80) **	0.98 (0.84–1.14)	0.79 (0.68–0.91) *	1.07 (0.86–1.34)
**Parent/guardian knowledge of location**				
Always or almost always know	(Reference)	(Reference)	(Reference)	(Reference)
Sometimes	1.86 (1.56–2.23) **	2.49 (2.12–2.91) **	2.05 (1.76–2.39) **	2.52 (2.06–3.08) **
Never or almost never know	1.14 (0.89–1.45)	1.39 (1.10–1.75) *	1.11 (0.89–1.38)	1.33 (0.99–1.78)
**Parental monitoring of media use**				
Yes	(Reference)	(Reference)	(Reference)	(Reference)
No	1.64 (1.47–1.83) **	1.60 (1.43–1.78) **	1.49 (1.35–1.64) **	1.60 (1.36–1.87) **
**Parental awareness of school activities**				
A lot	(Reference)	(Reference)	(Reference)	(Reference)
Fairly	1.63 (1.43–1.84) **	1.38 (1.20–1.57) **	1.41 (1.26–1.59) **	1.57 (1.29–1.92) **
A little	2.17 (1.86–2.54) **	2.01 (1.73–2.33) **	1.99 (1.73–2.28) **	2.21 (1.78–2.74) **
None	1.67 (1.21–2.30) *	2.75 (2.02–3.75) **	1.35 (1.01–1.82) *	2.71 (1.88–3.92) **
**Shared meals with parent/guardian**				
Daily occurrences	(Reference)	(Reference)	(Reference)	(Reference)
Other occurrences	1.56 (1.40–1.75) **	1.63 (1.46–1.81) **	1.51 (1.36–1.67) **	1.64 (1.41–1.91) **
**Weekend curfew monitoring by parent/guardian**				
Yes	(Reference)	(Reference)	(Reference)	(Reference)
No	0.91 (0.77–1.07)	1.40 (1.20–1.64) **	1.05 (0.90–1.21)	1.63 (1.33–1.99) **
**Parental expectation to disclose outings**				
Yes	(Reference)	(Reference)	(Reference)	(Reference)
No	0.60 (0.47–0.77) **	1.47 (1.14–1.91) *	0.86 (0.67–1.10)	1.36 (1.03–1.80) *
**Parental awareness of close friends**				
Considerable	(Reference)	(Reference)	(Reference)	(Reference)
Somewhat	1.29 (1.15–1.46) **	1.32 (1.18–1.49) **	1.17 (1.05–1.30) *	1.33 (1.13–1.58) *
Barely	1.36 (1.14–1.62) *	1.58 (1.34–1.88) **	1.33 (1.13–1.56) **	1.68 (1.35–2.09) **

EDs: energy drinks; AmEDs: energy drinks mixed with alcohol; OR: odds ratio; 95% CI: 95% confidence interval; * *p*-value: <0.05; ** *p*-value: <0.001. Models were adjusted for age, residence, and ethnicity.

## Data Availability

The original data presented in the study are openly available in SENDA at https://www.senda.gob.cl/estudio-observatorio/poblacion-escolar/ (accessed on 30 October 2025).

## Supplementary Materials

The following supporting information can be downloaded at: https://www.mdpi.com/article/10.3390/nu17213481/s1, Table S1: Prevalence estimates of Energy drinks consumption: Lifetime, Past-Month and Mixed Use with Alcohol in complete cases versus full sample.

## Abbreviations

The following abbreviations are used in this manuscript:

EDs	Energy drinks
ENPE	The 2023 Fifteenth National Study on the School Population of Chile
AmEDs	Energy drinks mixed with alcohol
SENDA	Chile’s National Service for the Prevention and Rehabilitation of Drug and Alcohol Use
PSUs	Primary Sampling Units
PPS	Probability proportional to size
SSUs	Secondary Sampling Units
OR	Odds ratio
95% CI	95% confidence interval
