# Supplementary material for: Prevalence and Determinants of Energy Drink Consumption Among Chilean Adolescents
Source: Nutrients. 2025 Nov 5;17(21):3481. doi: 10.3390/nu17213481 (PMC12608689; doi:10.3390/nu17213481)
Supplement: Supplementary file 1 [file nutrients-17-03481-s001.zip › nutrients-3927707-supplementary.pdf]

Supplementary Table S1. Prevalence estimates of Energy drinks consumption: Lifetime, Past-Month and Mixed Use with Alcohol in complete cases versus full sample.

Variable	Complete Cases (n= 45,042)	Full Sample (n = 49,211)	p-value[a]
**EDs Lifetime % (95% CI)**	71.0 (70.3- 71.8)	68.7 (68.0- 69.5)	**< 0.001**
**AmEDs Lifetime % (95% CI)**	23.0 (22.4- 23.7)	22.2 (21.5- 22.9)	0.10
**EDs Past-Month % (95% CI)**	46.2 (45.3- 47.0)	44.0 (43.2- 44.8)	0.26
**AmEDs Past-Month % (95% CI)**	10.1 (9.6- 10.6)	9.7 (9.2- 10.1)	0.73

ED: Energy drinks; AmEDs: Energy drinks mixed with alcohol; 95% CI: 95% confidence intervals; * p-value: < 0.05; **p-value: <0.001.

[a] Test of homogeneity
